# Investigation of argon plasma treatment on the structural, surface morphology and electrical properties of bovine cortical bone

**DOI:** 10.1038/s41598-025-19684-2

**Published:** 2025-09-24

**Authors:** A. M. Abdel Reheem, Omar S. Desouky, Nabila S. Selim, H. M. Abdel-Hamid, Seham M. El-Marakby

**Affiliations:** Radiation Physics Department, Radiation Applications Division, National Center for Radiation Research and Technology, Egyptian Atomic Energy Authority, Cairo, Egypt

**Keywords:** Bovine cortical bone, Cold plasma, Surface roughness, Relative permittivity and ac conductivity, Biophysics, Structural biology, Materials science

## Abstract

In this study, bovine cortical bone was investigated under plasma treatment process to investigate the potential for improvements in their structural characteristics. The bone specimens were treatment with low pressure argon cold plasma at different treatment times; 15, 30, and 45 min. Various techniques such as X-ray diffraction, scanning electron microscopy, surface roughness testing and automatic LCR Bridge were utilized to study the plasma-induced modifications on the structural and dielectric properties of the bone. SEM images revealed the elimination of some outer atoms from the bone surface during the 30-minute plasma ablation process, leading to more noticeable grain size of hydroxyapatite. XRD measurements confirmed the obtained results as mentoring the changes in crystallite size and strain parameters. As the treatment time approached 45 min, crystallite size increased, along with surface roughness parameters and relaxation time. These findings contribute to a better understanding of the microstructural and morphological changes occurring on the bone surface during cold argon plasma treatment.

## Introduction

Bone research is a dynamic field of scientific exploration that typically includes multiple studies. Most of these investigations were conducted to attain ideal bone qualities and adequate surface properties while optimizing the external architecture^1–4^. Other research has been undertaken to help induce new bone formation while maintaining the present bone structure^5,6^.

Plasma can be classified according to different types such as pressure: vacuum or atmospheric^7^. When the plasma produced under normal pressure UV radiation is produced. When the plasma produced with vacuum, the pressure is less than 0.1 torr the plasma composed of ions, electrons, neutral particles, and may be irradiated UV and free radicals, depend of the energy. These species interact with a biomaterial and affect its physical and chemical structure. The principle for this alteration is plasma etching, which only impacts the surface layers of the material, restricted to a particular depth of a surface layers, thus stimulating the mechanical removal of surface impurities through intense electrons and ion irradiation^8,9^. These alterations cause surface chemical changes, which affect optical and electrical properties of the bombarded materials^10,11^. Over the last ten years, there has been a tremendous increase in the use of low-temperature argon plasma to influence biological hard tissues for medical purposes^12,13^. Argon plasma can affect the surface structure and morphology of biomaterials and improve the blood circulation and integrated the tissue of implanted subcutaneous^14,15^. From a physicochemical point of view, plasma irradiation increases the surface energy and decreases the contact angle; promote the cell spreading^16^. Additionally, plasma irradiation activates the surfaces at the atomic and molecular levels, increasing the the hydrophilicity of the surfaces and enhancing the wettability, adhesion, sterilization and improved the surface properties^17^ and, consequently, improves the regenerative potential of the bone grafts^18–20^.

Bone allograft, coming from bone banks, provides an osteo-conductive scaffold for new bone formation. It can come in different forms such as cortical, cancellous, and cortico-cancellous. Its preparation requires treatment with ethanol in order to remove cellular material, followed by sterilization by gamma irradiation for bacterial, fungal, and viral suppression. Consequently, allografts lose some of their biological properties through their preparation processes. The initial interactions between cellular components and the allograft materials play an important role in the healing process around implants^21^. They are affected by the quality of the allograft surface. Plasma irradiation is used to increase cellular adhesion to the surface of polymeric materials^22^, since it can induce changes by introducing polar functional groups in polar and hydrophobic parts^23^. Plasma irradiation of cortical bone proved to effectively cause a morphological alternation consisting of increased roughness of the bone surface due to the formation of cavities that increase porosities. Argon cold plasma is used due to its high etching efficiency during which the mechanical removal of the surface contaminants^17,24^. Moreover, several studies have been performed to measure the dielectric properties of bones in both the low-frequency range^25,26^ and in the microwave frequency range^27,28^. While some of these studies only measured the dielectric properties of bone, some studies also investigated the relationship between the bone quality and dielectric properties.

The objective of the current study is to investigate the effect of low pressure cold argon plasma on the physical properties of cortical bone, such as structural, morphological and electrical properties, in order to improve its use in bone grafting and overcome the problems commonly encountered by patients during bone transfer procedures.

## Materials and methods

### Samples preparation

Fresh bovine femurs were obtained from a local abattoir, from male animal aged 2 years, and stored at −20 °C until use. A surgical knife is used to scrape the periosteum off the bone surface^29^. The bone is sliced into samples of 25 mm^2^ and 3 mm thickness with a bone saw, the length was aligned with the longitudinal direction and the thickness in the radial direction. The samples were cleaned in an ultrasonic bath in an aqueous solution of 1% Triton-X (nonionic detergent) for 30 min before being irrigated with 35 °C warm water. The samples were then steeped in 70% denatured ethanol at room temperature for 60 min, and then were irrigated with flowing 35 °C warm water^30^.

The samples are treatment with argon plasma using cold plasma source^31^ in the charged particles lab., National Center for Radiation Research and Technology. It consists of stainless steel cathode sphere and copper anode disc with inner diameter 10 mm. The anode and the cathode are insulated by Teflon. The working argon gas is admitted through a finely controlled needle valve. The irradiation conditions are used the discharge current 1.5 mA, cold plasma current is 2 mA and the energy is 4 keV and the treatment times are 15, 30, and 45 min, respectively. Also, the working pressure is 2 × 10^−3^ mbar, electron temperature equals 1.4 eV, and the electron density is 1.6 × 10^8^ cm^−3^.

### Characterization techniques

The structure of samples before and after argon plasma irradiation was studied by the X-ray diffraction technique Shimadzu XRD- 6000. A Scanning Electron Microscope (SEM) ZEISS Microscopy EVO 15, and the surface roughness tester 5110 T were used to investigate the morphological and surface roughness changes of the samples. A programmed automatic RLC bridge (HIOKI 3532 LCR Hi-Tester, Koizumi, Japan) was utilized to study the dielectric activity at frequency range of 1 Hz to 5 MHz using metallic stainless steel circular electrodes (1 cm in diameter) in a sandwich configuration to ensure good electrical contacts.

## Results and discussion

Bone is a multi-phase hierarchical structure made up of inorganic, organic, and water components. The organic fraction is primarily collagen, accounting for 20–30% of the total bone tissue and 90% of the organic component. Type I collagen accounts for 85–90% of total bone protein^32^. Non-collagenous proteins, lipids, and water (about 10%) are also found in bone. The main inorganic matrix component is hydroxyapatite crystal, which has a similar structure to calcium hydroxyapatite Ca_10_ (PO4)_6_ (OH)_2_ mineral and accounts for 60–70% of the bone tissue^33–35^.

### Crystallite size parameters calculation

In calculating the crystallite size, the obtained peaks at 2θ degrees in the XRD spectra have been determined using Debye-Scherrer formula^36^ as presented in Eq. (1):

1 $${\text{D}} = {\text{K}\lambda}/\beta {\text{cos}\theta}$$

Where K is the shape factor (0.9), ‘λ’ is wavelength of X-Ray (0.1541 nm), ‘β’ is the full width at half maximum (FWHM), ‘θ’ is the diffraction angle and ‘D’ is particle diameter size.

The interlunar spacing between the atoms (d) is calculated using Bragg’s Law in Eq. (2).

2 $${\text{2dsin}\theta} = {\text{n}\lambda}~\left( {{\text{n}} = {\text{1}}} \right)$$

The lattice strain (ε) can be calculated using the Eq. (3)^37,38^:.

3 $${\varepsilon } ={\beta }/{\text{4tan }}\theta$$

Figure 1 shows the XRD spectra of the bovine cortical bone for blank sample and argon plasma treatment samples. It provides information about the crystallographic structure, chemical composition, and physical properties of the specimens. From Fig. 1 the crystallite size was calculated using the diffracting plane (002) of hydroxyapatite at 2θ = 25.3°, since this miller index corresponds to the c-axis length which is the direction of maximum elongation^35^.The spectrum displays diffraction peaks at 2θ equals 25.3° which are specific for hydroxyapatite of the bone samples with a semi crystalline structure. As shown in Fig. 1, there is a shift toward lower angle as the time of exposure increase, which can be attributed to the strain change^37^.

**1:**
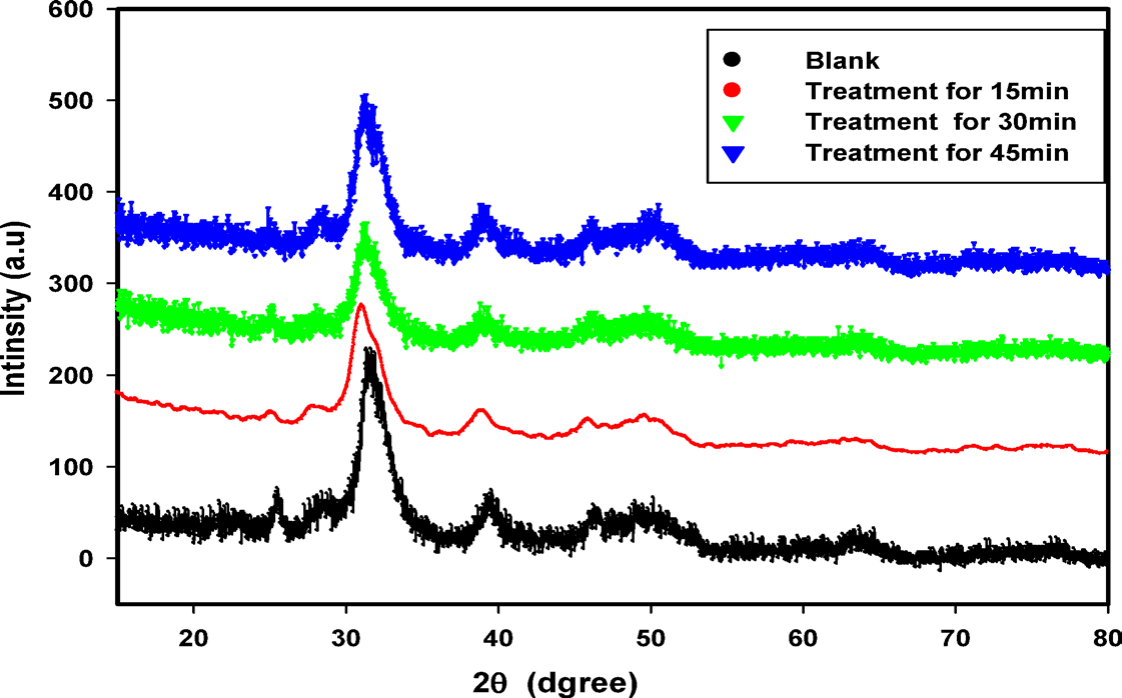
XRD patterns of cortical bone of different plasma treated samples; 15, 30, and 45 min’ treatment time.

**2:**
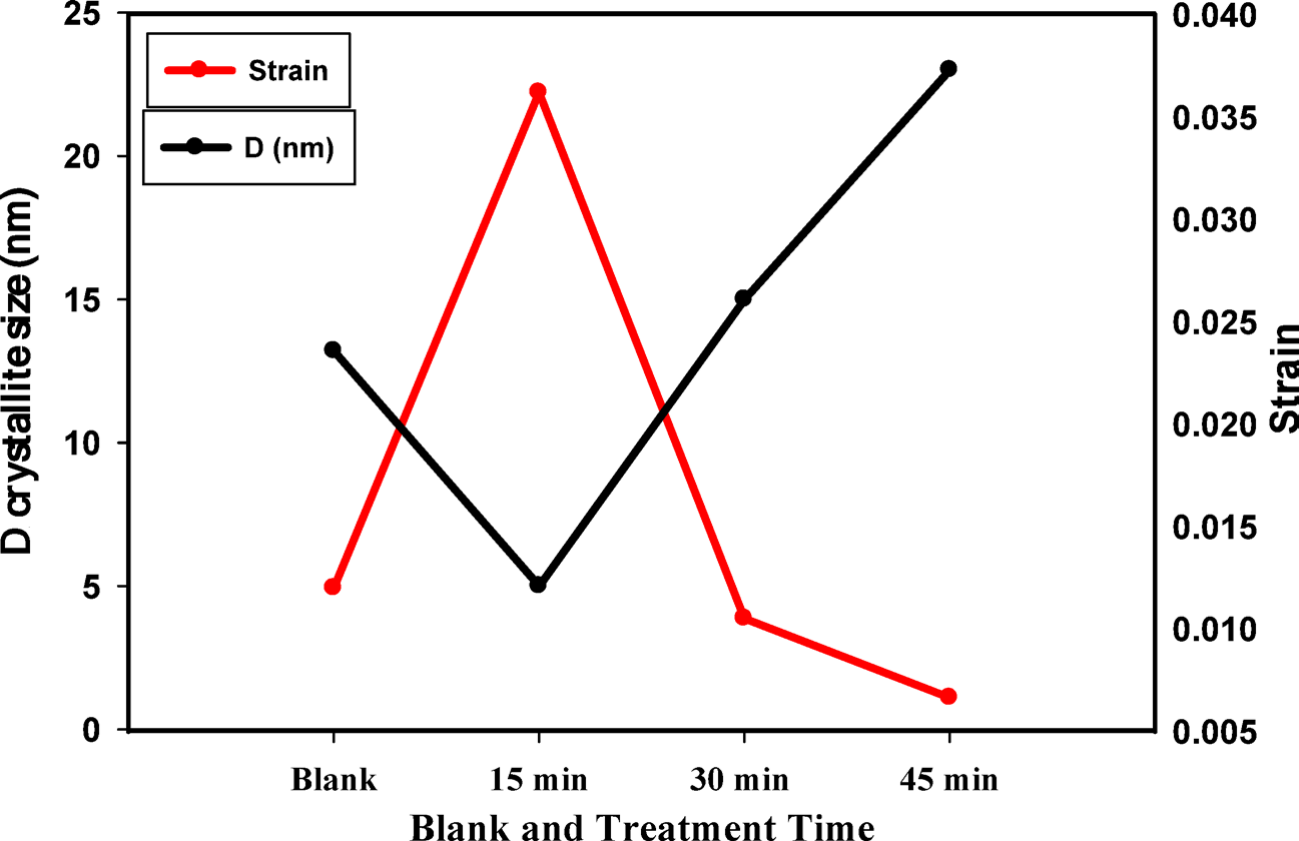
Effects of plasma treatment time on the crystallites size and the strain of bovine bones.

The calculated crystallite size, spacing (d) and strain for control and argon plasma treated samples are tabulated in Table 1. Figure 2 shows the relation between the crystallites diameter and strain for the bone control sample and the argon treated samples with different treatment times. It can be noticed that the crystallites diameter for the treatment sample at 15 min records 5 nm lower than both the samples irradiated at 30 and 45 min, respectively. The decreases in crystallite size lead to increase the surface area of the samples and increased the surface adhesion. In the same context, the strain shows an inverse behavior with crystallite diameter versus the treatment times (Table 1). These results are consistent with the results of the SEM images and the surface roughness data.

**Table 1:** Crystallite parameters crystal size of the control and different treated samples, obtained from XRD.

Sample	2θ (degree)	d (spacing) nm	D crystallite (nm)	Strain
Blank	25.47	0.349	13.2	0.012
15 min	25	3.62	5	0.0362
30 min	25.03	3.55	15	0.01062
45 min	25.11	3.54	23	0.00665

### Scanning electron microscopy

Figure 3a, b, c, and d show the microstructural changes in the cortical bone surface for both the control sample and the Ar plasma treated samples at various irradiation intervals; 15, 30, and 45 min, respectively. SEM is used with magnification 1kX for the samples before and after plasma treatment. Control sample shows an irregular dense, low porosity bone surface. The first treated sample, 15 min, shows a smoother surface with the emergence of multiple grooves as a result of the argon plasma treatment process. At the treatment time of 30 min, the surface of the sample shows more cleaning process with eliminating more outside atoms, indicating that the plasma ablation process has occurred. As the collagen layer was removed, the grain size of the hydroxyapatite crystals on the surface bone became apparent. As the treatment time reaches 45 min, the quantity of hydroxyapatite grains on the surface increases.

**3:**
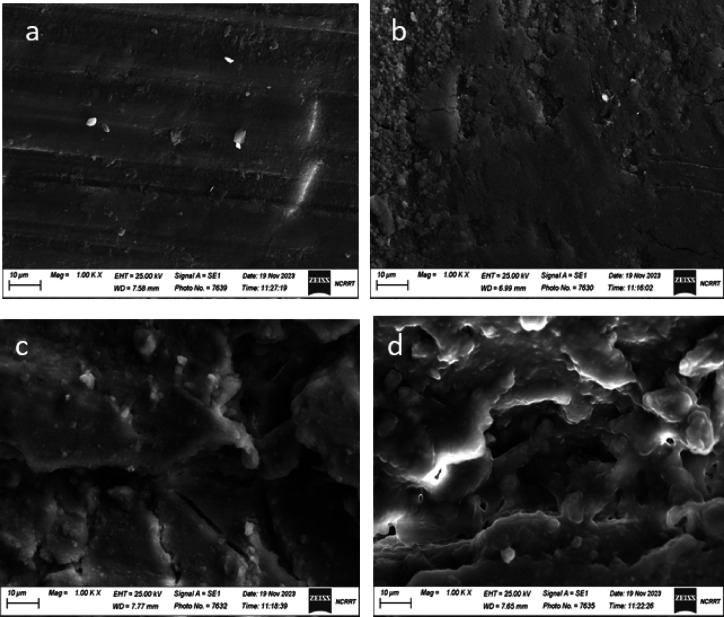
SEM images for the effect of plasma on the surface of bovine bone: (**a**) blank (untreated), (**b**) plasma treated for 15 min (**c**) plasma treated 30 min., and (**d**) plasma treated for 45 min.

### Surface roughness measurements

Microstructural differences were observed via the characterization of surface roughness, for the studied samples. This section focuses on the bone surface topography (surface roughness). The bone surface has imperfections of many orders, ranging from form deviations to irregularities of interatomic distances. The surface roughness tester 5110 T was used to measure the samples’ surfaces for Blank sample and argon plasma treatment samples for 15, 30 and 45 min, respectively.

R_a_ and R_z_ are the most commonly utilized parameters used to numerically describe the appearance of surface roughness. These metrics are solely height descriptive. R_a_ is the arithmetic mean of the roughness profile’s deviations from the mean line, and R_z_ is the average height difference between the five highest peaks and five lowest troughs within the profile,

Figure 4 shows the relation between the two amplitude parameters: R_a_ and R_z_ values. The obtained results for R_a_ were 0.18, 0.085, 0.25, and 0.26 μm, meanwhile, R_z_ were 0.18, 0.09, 0.22, and 0.27 μm for untreated and plasma treated samples at the mentioned treatment times. As illustrated in Fig. 4, the bone surfaces are altered by plasma treatment. The sample that is treated for 15 min of argon plasma reduced the roughness of the bone surface. This decrease is caused by the ablation of the outer surface layer. The roughness increases again after 30 and 45 min of treatment, which could be due to crystal or bond cleavage. The results are matching with the SEM images. The surface roughness allows for vascularization and the ingrowth of new bone^39^.

**4:**
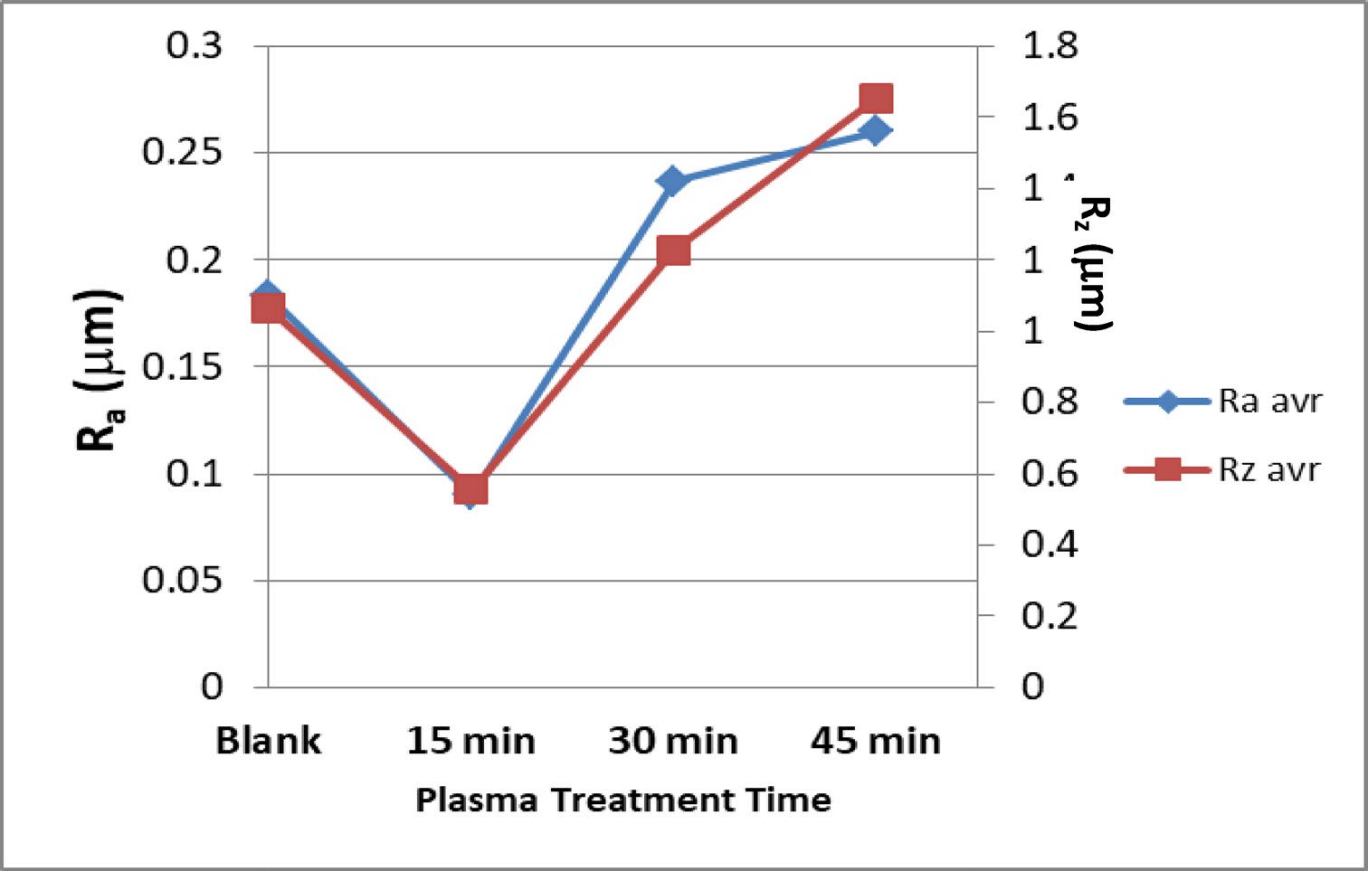
Effects of plasma treatment time on the surface roughness parameters; R_a_ and R_z_.

### Dielectric properties measurements

Bone tissue has been identified as dielectric material; Non-conductive materials capable of exhibiting polarization of negative and positive charges when an external electric field is applied^40^. The dielectric properties can be characterized by: relative permittivity, dielectric loss, AC conductivity (S/m) and relaxation time (s). These parameters are changed due to separation of hydrogen bonds between collagen and hydroxyapatite (HA) upon applied an electrical field. The bone composition, especially ions free to move and the bound electrical charges within organic bone matrix, significantly contributes to these electrical and dielectric parameters^41^. The permittivity, as a measure of the polarizability and related to the structural arrangement of the polarized groups, does not occur instantaneously, and the associated time constant is called the relaxation time (s). The dielectric loss is estimated by the total area under the loss curve, which is proportional to the total concentration of dipoles in the tissue and their dipole moment, irrespective to their distribution of relaxation times^42^. The AC conductivity (S/m) depends on the dynamical transport of the structural ionic and polar groups through the tissues. Several studies have shown that electrical properties, specifically dielectric properties of biomaterials are of great interest to understand bone healing mechanism^43^.

The measured parameters are the capacitance C, resistance R and conductance G, from which the permittivity ε’ is calculated^44^.

4 $${\text{C}} = {\text{A}}\upvarepsilon^{\prime } \upvarepsilon_{{\text{o}}} /{\text{d}}$$

Where A is the area of the electrode and d is the distance between the two electrodes and ε_0_ is the vacuum permittivity (F/m). The permittivity can be expressed in complex quantity as:

5 $$\upvarepsilon * = \upvarepsilon ^{\prime } {-}{\text{j}}\upvarepsilon ^{{\prime \prime }}$$

The real part represents the permittivity constant and is given by:

6 $$\varepsilon ^{\prime } = \varepsilon _{\infty } + \frac{{\varepsilon _{s} - \varepsilon _{\infty } }}{{1 + \omega ^{2} \tau ^{2} }}$$

Where ε_s_ is the limiting low frequency permittivity, and ε_∞_ is the permittivity value at the end of the dispersion, *τ *is the relaxation time and the imaginary part ε” (the dielectric loss):

7 $$\varepsilon ^{{\prime \prime }} = \frac{{\left( {\varepsilon _{s} - \varepsilon _{\infty } } \right)\omega \tau }}{{1 + \omega ^{2} \tau ^{2} }}$$

The AC conductivity σ (S/m) can be calculated using Eq. (8).

8 $${\sigma} = {\text{G d}}/{\text{A}}$$

Also; the relaxation time (s) is calculated using Eq. (9)

9 $$\uptau _{{\text{r}}} = {\text{1}}/\upomega _{{\text{r}}}$$

In the present study, the variation of electrical and dielectric properties across the low frequency range (1–100 kHz) for both untreated and plasma treated bone samples among the different duration times of treatment were plotted in Figs. 5, 6, 7 and 8. The following points have been noted:

1. In order to evaluate the variation in in the relative permittivity values, a common frequency range (1–10 Hz) is chosen, as a first interval, for all samples as shown in Fig. 5. It is found that the relative permittivity values of all treated bone samples are higher than that in the untreated bovine sample. However, for the 30 min treated sample, the data exhibit the highest value over other treated samples, reaching a value double that of the control sample but with extended frequency range (1–50 Hz), as a second interval. Then, both issues show a steeply decrease in the values down to the unity with increasing frequency up to 1 kHz. Then, no variation is determined, as the frequency increases up to 100 kHz.
2. The loss factor increases with increasing the treatment time up to 30 min. then it gets lower, as the treatment time reaches 45 min as shown in Fig. 6. Moreover, loss factor in no case exhibits “pronounced” peak which is at ∼ 50 Hz, for all bone samples except for the case of 30 min time, the peak is at ∼ 100 Hz, which might be associated with a single relaxation time. The increasing values may be due to bonds breaking, which increase the amount of defects on the bone surface. These imperfections result in an increase in the number of dipoles in the samples, which intensifies the polarization.
3. The variation in conductivity values vs. the frequency is shown in Fig. 7 for both the blank and the treated bone samples. Across the frequency range (10–100 Hz), the conductivity gradually increases, then, at higher range (100–100 kHz) it becomes saturated. For example, at frequency (1 kHz), The measured conductivity, for the 30 min treated sample is about 23 × 10^−8^ S/m, while it is 7 × 10^−8^ S/m for the 45 min treated sample. This reflects the increase in the free ions released after Ar plasma treatment, which became depleted after 45 min treatment resulting in decrease in the conductivity.
4. The variation in relaxation time values for both the control and plasma treated samples at the different treatment times is plotted in Fig. 8. Results show that the 30 min treated sample has a lowest relaxation time; 2.5 × 10^−3^s while that for the control sample is 8 × 10^−3^s. This decreasing value in the relaxation time reflects the increase in the electric charge and/or increase in the size of the polarized part moving in the applied electric field. This effect is depleted as the treatment time increases to 45 min. In this case, the polar groups tend to decrease as the relative permittivity and dielectric loss decrease associated with the increase in the relaxation time^45^. It is clearly noticed the significant dependence of the relaxation time on both the electric charge and the size of the polarized groups. These effects can be attributed to the Ar plasma treatment which may result in unfolding of the quaternary and tertiary structures of the protein contents in bone tissues resulting in increase in the concentration of the polar groups to respond to the applied electric field. Moreover, there is an increase in the well-known reactive oxygen species (ROS) that are produced when the surface of the bone reacts with argon plasma.

**5:**
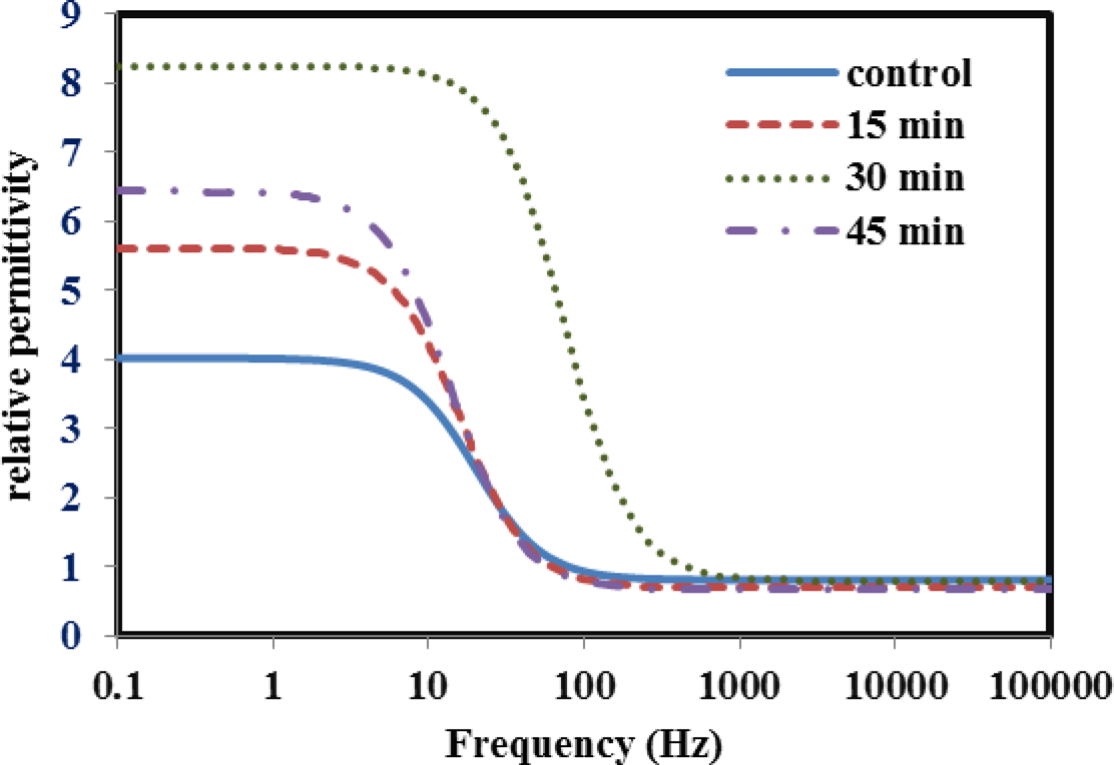
Frequency dependence for Relative permittivity of the control and argon plasma treated samples at different times; 15, 30 and 45 min.

**6:**
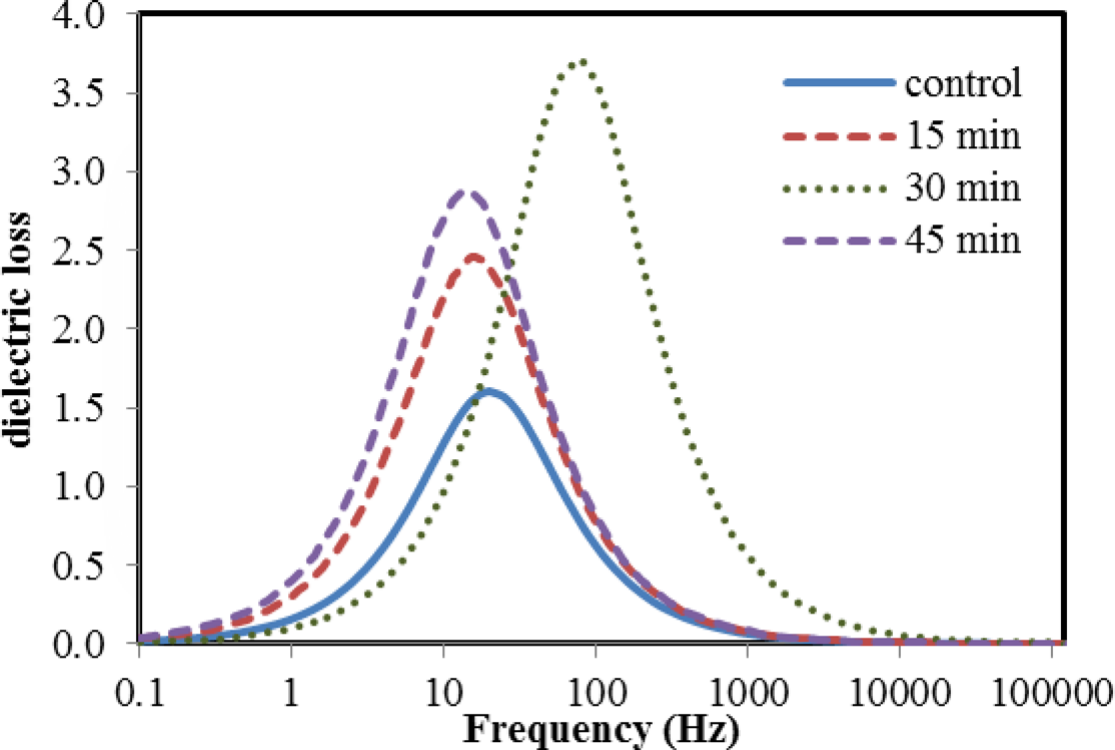
Variation of the dielectric loss with the frequency for control and argon plasma treated samples at different times; 15, 30 and 45 min.

**7:**
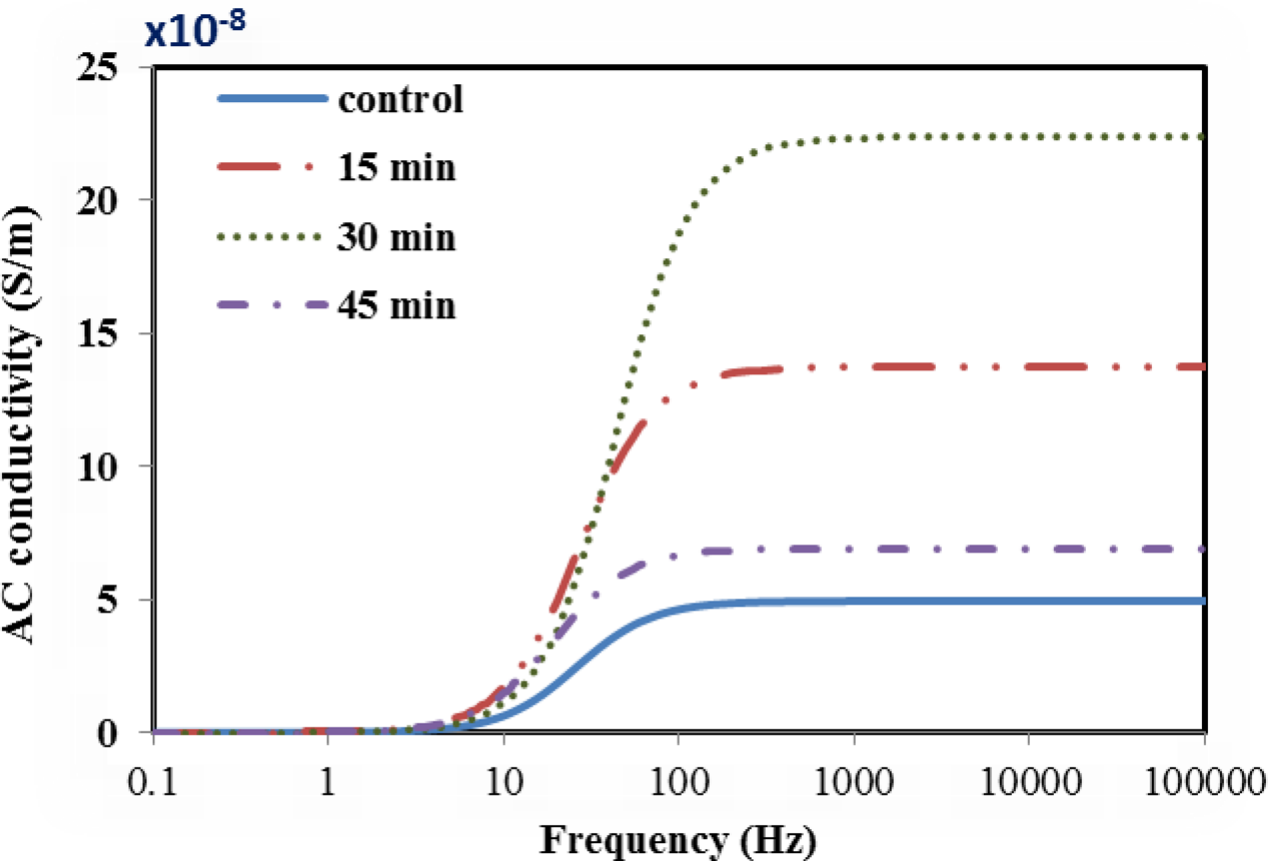
Frequency dependence of AC conductivity (S/m) for the control and plasma treated samples at different times; 15, 30 and 45 min.

**8:**
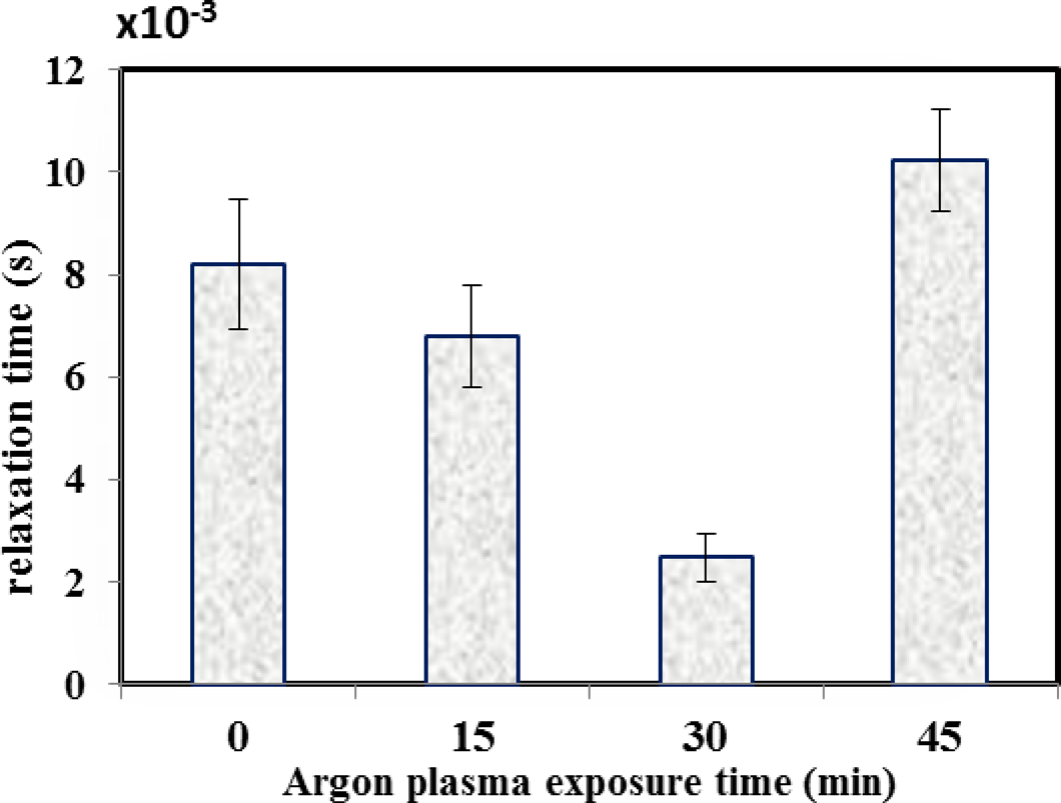
Variation of the relaxation time (s) for control and argon plasma treated samples at different times; 15, 30 and 45 min.

## Conclusion

This study demonstrates that low-pressure argon cold plasma treatment enhances the structural and electrical properties of bovine cortical bone, particularly at treatment durations up to 30 min. The findings indicate that plasma treatment improves dielectric properties, evidenced by increased relative permittivity and electrical conductivity, alongside notable microstructural modifications. SEM and XRD analyses reveal that plasma treatment alters surface morphology and crystallite characteristics, suggesting a correlation between treatment time and structural changes. While 30 min of treatment yields optimal results, extending the duration to 45 min leads to contrary changes in crystallite size and surface roughness, with diminished improvements in electrical properties. Overall, this research provides valuable insights into the potential applications of plasma treatment in enhancing biomaterials, paving the way for future studies on optimizing treatment parameters for improved performance in biomedical applications.

## Data Availability

All data generated or analysed during this study are included in this published article.
